# Virtualized Gamified Pharmacy Simulation during COVID-19

**DOI:** 10.3390/pharmacy10020041

**Published:** 2022-03-26

**Authors:** Denise L. Hope, Gary D. Grant, Gary D. Rogers, Michelle A. King

**Affiliations:** 1 School of Pharmacy and Medical Sciences, Griffith University, Gold Coast, QLD 4222, Australia; g.grant@griffith.edu.au (G.D.G.); michelle.a.king@griffith.edu.au (M.A.K.); 2 School of Medicine, Deakin University, Geelong, VIC 3220, Australia; g.rogers@deakin.edu.au

**Keywords:** virtualization, pharmacy education, gamification, simulation, experiential learning, active learning

## Abstract

Extended and immersive gamified pharmacy simulation has been demonstrated to provide transformative learning in pharmacy education, preparing graduates for real-world practice. An international consortium of universities has implemented local adaptations of the Pharmacy Game into their curricula. From early 2020, pharmacy academics modified the delivery of gamified simulation in response to the COVID-19 pandemic, while still aiming to deliver the important learning outcomes of enhanced communication, collaboration, confidence and competence. Australian universities went into full lockdown from March 2020, and the critical gamified simulation at Griffith University was delivered entirely virtually in 2020. An array of synchronous and asynchronous approaches and software platforms was employed, including Microsoft Teams, Forms and Stream plus the online interview platform Big Interview. These allowed for the simulation activities, including dispensing, counselling and clinical cases, to be conducted by students online. In 2021, Griffith University conducted hybrid delivery of its Pharmacy Game, balancing student participation both in person and online. Microsoft Power Apps was added to the hosting platform to enhance the simulation interface, and Power Virtual Agent artificial intelligence chatbots, with natural language processing, were used to enable asynchronous clinical interaction. The combination of learning technologies provided the means to deliver successful gamified simulation in the virtual and hybrid environments while still achieving outstanding learning outcomes from the capstone activity. This paper details the technologies used to virtualize the Australian Pharmacy Game and the analytics available to educators to assess student participation, engagement and performance.

## 1. Introduction

The Pharmacy Game (https://pharmacygame.education/, accessed 9 February 2022) is an extended and immersive gamified simulation developed at the University of Groningen, the Netherlands [1,2]. It has been adapted and implemented in the pharmacy curricula of a consortium of international universities to deliver core and aspirational learning outcomes, including enhanced confidence, competence, collaboration and communication [3]. The gamified simulation traditionally involves student teams competitively managing their own in-person pharmacies over an extended period of weeks, during which time students assume the role of autonomous pharmacists in all professional and clinical decision-making. Team tasks include medicines dispensing and counselling, pharmaceutical care plans and regular consultations with standardized patients and simulated health professionals. The authentic nature of such simulation enables students to focus on patient-centered care, applying their knowledge and practicing professional skills in a safe learning environment, without any risk of actual patient harm [4,5]. The gamified aspects of the simulation motivate and engage students, with the application of continuous and competitive scoring providing immediate feedback on tasks [6]. Consequences of practice are recognized with optimal professional and patient outcomes rewarded through positive scoring, and potentially illegal, unprofessional or harmful behaviors penalized through negative scoring [7].

The Griffith University adaptation of the Pharmacy Game, PharmG, has been delivered as a capstone activity for final semester students since 2016 to prepare graduates for pharmacy internship and real-world professional practice [8]. Research undertaken by Griffith University on experiential learning in the gamified simulation confirmed its ability to support teamwork and collaborative learning [9]. Analysis of student participants’ reflective journals revealed the transformative capacity of the Pharmacy Game to influence their professional identities, patient-centeredness and future practice [9].

Prior to the COVID-19 pandemic, all consortium universities conducted their adaptations of the Pharmacy Game as fully immersive, in person simulations [3]. However, from March 2020, Australian universities closed due to the pandemic, which required complete transformation of all teaching and learning activities into the online space, as was true for much of the world [10,11]. The rapid switch to emergency remote teaching was combined with the acknowledgement that pharmacy educators “have a collective responsibility to prepare students to be practice-ready and team-ready for now and for the next global pandemic” [12].

The PharmG gamified simulation is a critical component of the Griffith University pharmacy curriculum. In 2020, it was modified to be delivered as a fully virtual activity, aiming to provide the essential learning outcomes of enhanced communication, collaboration, confidence and competence. By 2021, there was restricted return of students to Australian campuses with limitations on venue capacities and distancing requirements, which necessitated a hybrid delivery of the gamified simulation in the second year of the pandemic. This paper details the iterative adaptations made to virtualize the gamified simulation, the technologies used and the analytics available to educators to assess student participation, engagement and performance.

## 2. Virtualization of Gamified Simulation

**Figure 1:**
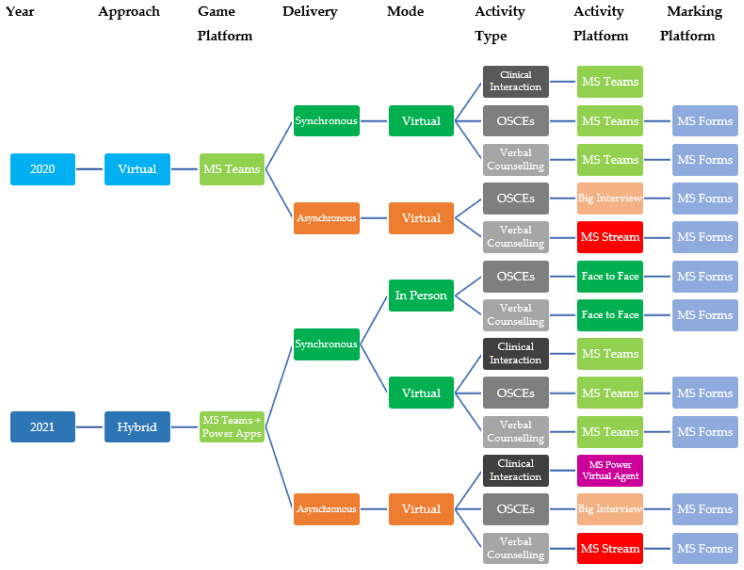
Approaches and software platforms utilized in 2020 virtual and 2021 hybrid gamified simulation delivery.

Virtualization of the gamified simulation was conducted using an iterative, design-based approach [13]. Due to the university lockdown in 2020, the only option for delivery was fully online, but academic reflection and student feedback then informed the iterative changes and improvements to deliver the hybrid model in 2021. Virtualization involved an array of synchronous and asynchronous approaches and various software platforms (Figure 1). In each year, student participants of the PharmG simulation were provided with a two-hour online briefing lecture in advance of their participation. All aspects of the activity were discussed, and students were given an extensive walkthrough of the various learning technologies that would be used. At the close of each year’s extended simulation, a three-hour debriefing session was conducted with all participants. In 2020, the debriefing was conducted online, whereas in 2021 the debriefing was conducted in person, with remote students connected to the event online. Institutional ethical approval for research on the gamified simulation was obtained from the Griffith University Human Research Ethics Committee (2016/594).

### 2.1. Stage 1: 2020 Virtual Delivery

Microsoft Teams [14] was identified as the optimal platform upon which to deliver the virtual PharmG simulation in 2020. Students and academic staff had varying previous exposure to Teams, as part of the Microsoft 365 suite of software products [15]. Teams provided a communication platform that allowed creation of a broad team for the simulation that comprised separate channels for each of the eight student teams. The academic game management team also had a unique channel (PharmG Headquarters), in which all files were stored and staff could privately plan and communicate. Students could chat and share files within their team channel and with the academic management team. Students could also contact each other or the simulated prescribers in the game by making audio or video calls through Teams. End-of-day submissions of dispensing labels and patient repeat forms, and any other assignments, were completed by teams uploading files to their relevant channels.

Daily prescriptions and clinical cases were shared as PDF files to each channel, and students completed their prescription assessment, evaluation and counselling in Microsoft Forms templates [16], which were adapted from previous paper-based templates. At the close of each game day, the Forms templates were downloaded to Microsoft Excel for each prescription case and then uploaded to the management channel of Teams, which allowed academic staff and supporting pharmacists to assess the individual prescription cases on the following days.

Prior to COVID, regular verbal prescription counselling was completed both face-to-face and via audio recordings. The mix of synchronous and asynchronous verbal counselling was continued during virtualization using several platforms. Live, synchronous counselling was planned and delivered by students on a Teams video call to a simulated patient who was joined by an assessor, or to an assessor who also assumed the role of simulated patient. The Teams calls could also be recorded if desired. Asynchronous medicines counselling was completed each day by recording of video counselling directed to the patient, which was uploaded by teams to their Microsoft Stream [17]. All verbal counselling was assessed using Forms, with each case’s former paper-based or OSCE-Eval for iPad [18] marking rubric being converted to Forms.

Objective structured clinical exams (OSCEs) were also completed both synchronously and asynchronously. Synchronous OSCEs were unannounced and were initiated by a simulated patient and/or assessor video calling the team, which students were instructed to treat as if a patient had walked into their virtual pharmacy. The OSCEs could be recorded if necessary. Asynchronous OSCEs were also completed each day using the Big Interview platform [19], which was originally designed for asynchronous video interviews but repurposed in this context for virtual OSCEs [20]. Teams were provided with an access code for the daily cases. Big Interview OSCEs involved students accessing and responding to sequential pre-recorded videos of patient case scenarios. Their targeted patient questioning and counselling videos were recorded in the platform in response to each patient interaction. Once completed, the game manager distributed the links to teams’ video responses to academic and pharmacist colleagues for assessment. Additional details of this adaptation have been published elsewhere [20]. As with other activities, marking rubrics were converted to Microsoft Forms templates. Once each verbal counselling or OSCE case was completed and assessed for all teams, the game manager downloaded Forms results to Excel and disseminated scores.

Clinical cases were transitioned from paper-based to online using SharePoint Pages [21] added to the Teams site for each team. Clinical interactions between students and simulated patients or simulated healthcare professionals were conducted virtually using Teams chats and video calls. Student-prepared pharmaceutical care plans were recorded in a Microsoft Form for review and marking.

**Figure 2:**
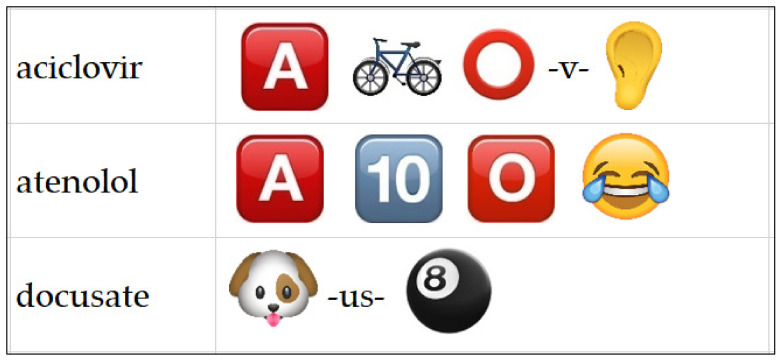
Exemplar drug-name-emoji challenge delivered in Microsoft Teams.

Given the virtual delivery of Pharm G in 2020, the management team created several engagement activities to involve students in a fast-paced and competitive manner during the simulation. These included episodic and timed release of fun tasks, such as tablet or capsule identification challenges and drug-name-emoji challenges, in which teams had to decipher a drug name from the emojis (Figure 2). These were prepared in advance of the simulation to encourage students’ online participation and engagement.

Griffith University was the first of the Pharmacy Game consortium of international universities to conduct their gamified simulation during the COVID-19 pandemic in 2020. The management team of PharmG invited British and European colleagues to visit the simulation in Teams, where they were given a virtual tour of the site and pharmacy team channels. The technological approaches that were developed and employed, and the learning that had occurred, were disseminated to colleagues to help inform their planning for their respective Pharmacy Game deliveries during the pandemic. Ultimately, only the Australian PharmG simulation was fully virtual in 2020 due to university closure, whereas the universities based in the United Kingdom and Europe delivered hybrid simulations in 2020.

### 2.2. Stage 2: 2021 Hybrid Delivery

Hybrid delivery in 2021 involved three students from each of the eight simulation teams being on campus each game day. Student teams managed their own rosters for in-person attendance. Some students were located interstate or overseas and unable to attend campus at all, due to border restrictions/closures, but the hybrid delivery provided flexibility to enable their involvement every day.

**Figure 3:**
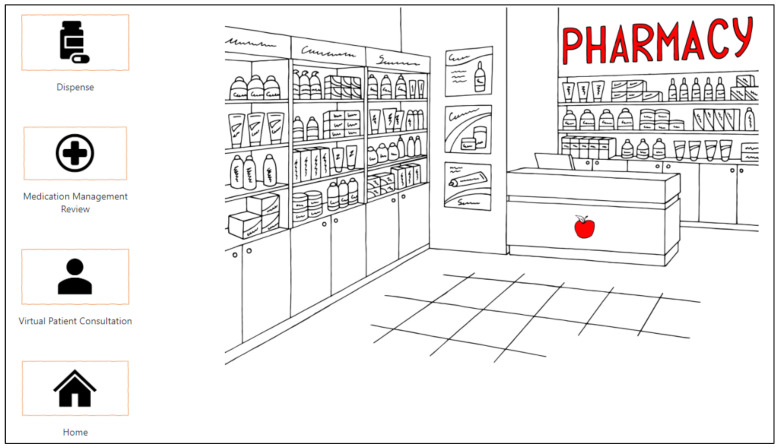
Team entry point to the Power Apps interface in Teams.

Teams was again used as the base platform for PharmG, with eight separate channels created for the student teams, one for the game Headquarters and the General channel where all participants could communicate. However, iterative enhancements were implemented to improve the simulation interface using the Microsoft Power Platform tools [22], which included Microsoft Power Apps [23]. The latter was used to create a smoother interface between participants and the virtual game setting (Figure 3).

**Figure 4:**
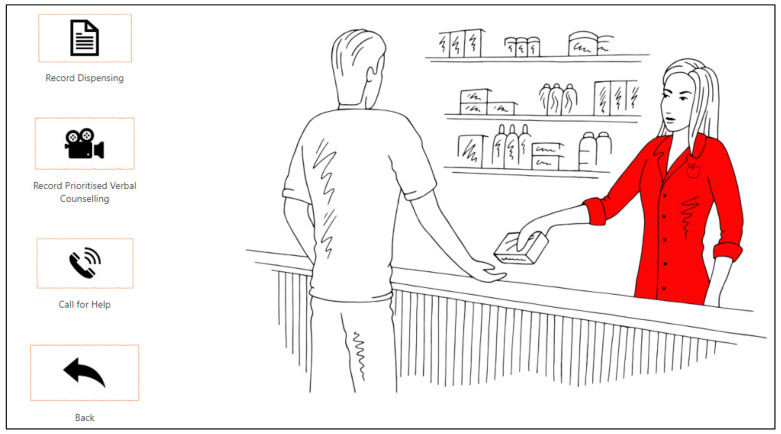
Individual patient options in the Power App.

Daily prescriptions were automatically and sequentially released, with individual patient profiles created that often included additional clinical data. This automated the workload distribution as game managers no longer needed to distribute prescriptions or clinical cases as PDF files, as they were embedded inside the Power App (Figure 4).

**Figure 5:**
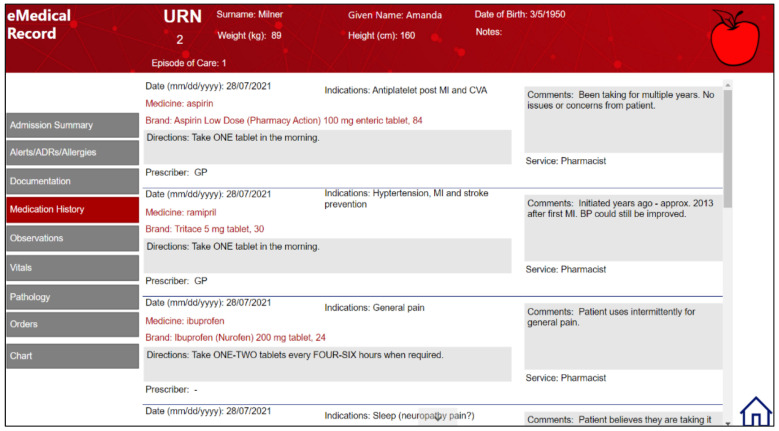
Electronic medical record developed using Microsoft Power Apps.

In 2021, Microsoft Power Apps was again used to deliver virtual clinical interactions and pharmaceutical case plans. It was also used to develop an electronic medical record system, which allowed for virtualization of clinical cases and display of real-time patient information for student review (Figure 5). Each team was provided with a secure, unique database so that information entered was only viewable by them and the management team. Student teams prepared and entered pharmaceutical care plans and interventions directly into the electronic medical record system, which was monitored and marked remotely by supervising staff.

**Figure 6:**
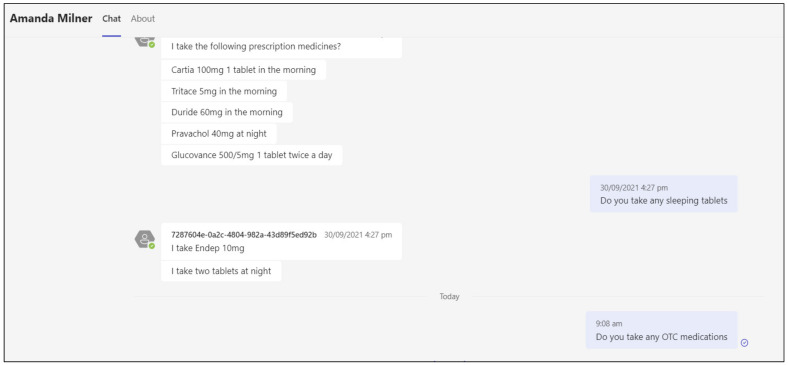
Example interaction between live student and Power Virtual Agent patient (Amanda Milner) chatbot.

A further iterative enhancement during hybrid delivery in 2021 was the use of Power Virtual Agents for Teams, which are artificial intelligence (AI) chatbots with natural language processing [24]. The chatbots were used for asynchronous clinical interactions between students and simulated patients or medical professionals. The availability of these tools allowed students to complete best practice medical histories for patients encountered as part of the clinical case and pharmaceutical care planning activities (Figure 6).

The PharmG structure of activities for hybrid delivery involved a mix of synchronous and asynchronous, in-person and virtual tasks every day. On campus students were exposed to in-person verbal counselling, live OSCEs and patient and prescriber consultations. Virtual students were able to complete live virtual verbal counselling in Stream, live synchronous OSCEs in Teams and asynchronous OSCEs in Big Interview. All students completed dispensing templates in Forms with the on-campus students using dispensing software to generate patient medicine labels and repeat forms for all cases.

The academic staff, pharmacists and support staff involved in managing the daily activities and assessments in PharmG had both an on campus and a virtual presence in the game. As most assessments were marked in Forms, there was flexibility as to how and where the virtual assessments were conducted. The on-campus cases were assessed in Teams with staff accessing the software from dedicated iPads.

## 3. Results and Observations

The 2020 virtual and 2021 hybrid deliveries of the gamified simulation succeeded in achieving the intended learning outcomes. This section presents data related to each delivery and describes the analytics available to educators to assess student participation, engagement and performance using these platforms.

### 3.1. Stage 1: 2020 Virtual Delivery

Eight online teams, comprising 65 students in mixed Bachelor of Pharmacy (BPharm) and Master of Pharmacy (MPharm) teams, completed the virtual PharmG simulation in 2020. The BPharm students (n = 40) participated full-time (every day for three weeks), and the MPharm students (n = 25) participated part-time (two days per week for three weeks).

Online student engagement and activity were firstly monitored by academic staff involved in the daily running of the simulation. When audio or video calls were made to Teams channels for each pharmacy, staff could observe which students were online and available for consultation. Audio and video calls were sometimes conducted with multiple or all members of a team for a particular debriefing or provision of feedback.

**Figure 7:**
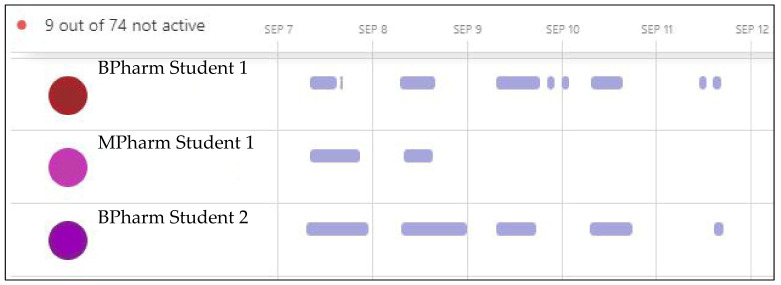
Teams Insights analytics on daily student engagement with Teams.

Additionally, educators could monitor individual student engagement with Teams using the Insights analytical tool to report on digital activity [25]. Insights allowed for observation of individual time active in the site (Figure 7), and it was easily identifiable if students were not actively participating, as only active time in the platform is visible. The Spotlights function of Insights provided an overview of activity and meeting participation and could identify individual students that were late, absent or inactive.

**Figure 8:**
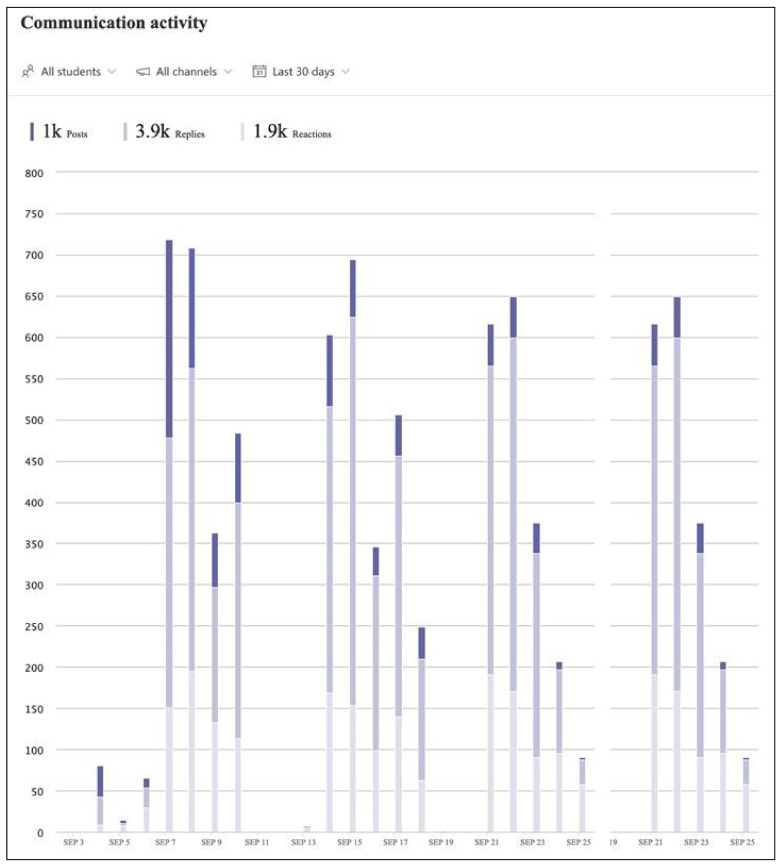
Communication activity reported for all students in Teams.

Figure 7 is an example of the automatically generated output displaying participants’ active time in Teams. The example reveals the expected engagement from the full-time students, although BPharm Student 2 was actively engaged with their pharmacy team for a longer duration on the first two days. Figure 7 also displays the anticipated part-time activity of the MPharm student, whose expected attendance was for two days only. Hovering over the bars of individual students’ engagement would further reveal which channels they had visited, how many messages they replied to, the duration of meetings they attended and who had organized the meetings. It was also possible to report Insights data by individual student to see what digital activities each had engaged in while logged into Teams, including Teams chats, meeting attendance and file opening and exchange. The communication activity data reported how engaged students were with posts, replies and reactions [26]. These data could be displayed for different time intervals and by individual student or specific channels, which would reveal each pharmacy team’s communication frequency. Figure 8 is an example of the automatically generated communication activity output in Teams. The example displays the communication activity for all students across the three weeks of the virtual game in 2020, displayed as number of posts (dark bars), replies (medium bars) and reactions (pale bars). Across the duration of the simulation, more than 1000 posts were created, generating over 3900 replies and 1900 reactions (Figure 8).

#### 3.1.1. Student Feedback on Virtual Delivery

In the final debriefing, students reported positively on the teamwork aspects of the simulation but expressed some degree of frustration at being isolated from their team members and unable to attend campus or work together in person. Anonymous student feedback on the virtual simulation was obtained from free text comments on course evaluation reports for the semester that delivered PharmG.

Positive exemplar comments from the virtual simulation include:

*The Game! It was an honest way of finding out where our skillsets were albeit rather stressful. I also enjoyed [academic staff] attempts at trying to engage us throughout the virtual world.*

(Student A, Student Evaluation of Course 2020)

*PharmG was an absolutely amazing experience. Gave everyone a chance to experience a somewhat real pharmacy experience. More than anything gave (at least me) a lot of valuable skills required for everyday life. Even though PharmG was required to be delivered online, there was no disadvantage. All the staff involved worked very hard to transform this activity and did an amazing job.*

(Student D, Student Evaluation of Course 2020)

Negative exemplar comments include:

*The virtual experience wasn’t well prepared for, but then again no one expected a pandemic.*

(Student G, Student Evaluation of Course 2020)

*PharmG got a bit repetitive and it felt like I was just sitting at my desk writing up dispensing templates day after day, which got a bit exhausting.*

(Student I, Student Evaluation of Course 2020)

#### 3.1.2. Staff Reflection on Virtual Delivery

The initial workload to virtualize the gamified simulation in 2020 was enormous. Several staff were involved in the planning and development of tools and processes required to conduct a virtual simulation. G.D.G. led the decision-making and adaptation of the technologies, building the pharmacy team channels in Teams, creating patient profiles and embedding daily prescriptions and developing the interface for clinical interactions and pharmaceutical case planning tasks. D.L.H. was responsible for converting all of the previous face-to-face OSCEs and patient cases to virtual and digitizing all of the previously paper-based dispensing templates and marking templates in Forms.

Preparing OSCEs for asynchronous completion in Big Interview required modifying each previous actor case into sequential scripts that could be recorded in short, progressive videos from the patient perspective, to which students would respond. Twenty-eight cases were recorded with four or five videos each by various staff and colleagues, from the pharmacy and other disciplines. This required many hours of time to convert scripts, recruit simulated patients, record and upload videos then assemble sequential cases in Big Interview.

Interaction with students was limited to virtual, through live contact in Teams and during synchronous cases and OSCEs. In addition to the Teams analytics described, staff could monitor individual student participation through observation of assessment contributions, as all submissions and completions in software platforms identified the students.

Response rates to student evaluations of the courses that hosted PharmG were the lowest ever recorded in 2020 (17.5% and 20.0%), potentially due to the digital fatigue experienced by students during the pandemic. The course evaluations were still positive but lower than usual for the two courses (4.0 and 4.1 out of 5, compared with 4.7 and 4.9 in 2019). The participating student cohort were on the cusp of graduation and appeared to be angry and frustrated at not having any face-to-face interaction with their fellow students during their final semester, and this was reflected in the course evaluations.

Ultimately, staff were satisfied that the learning outcomes of the gamified simulation were met, even with the challenges of the fully virtual delivery of the activity in 2020. While time consuming, the benefit of the initial workload completed in 2020 and inaugural delivery of the virtualized simulation meant that that all digital resources were then readily available, and iterative improvements could be implemented for hybrid delivery in 2021.

### 3.2. Stage 2: 2021 Hybrid Delivery

Eight hybrid teams, comprising 70 BPharm students, completed the hybrid PharmG simulation in 2021. The simulation was delivered one day per week across the semester, as opposed to the previous block delivery. As such, the MPharm students were unable to participate in the main delivery of PharmG due to timetable incompatibility. The majority of BPharm students (n = 64) were able to rotate through rostered in-person participation. Six students were interstate or overseas and due to border restrictions/closures were unable to attend campus.

**Figure 9:**
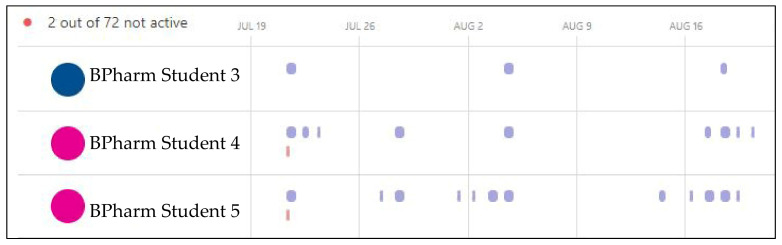
Teams Insights analytics on weekly student engagement with Teams.

Students’ engagement, digital activity and in-person conduct was monitored by academic staff through a combination of observation and Teams Insights. With the delivery of PharmG undertaken one day per week in 2021, the Insights report reflected that information. However, Figure 9 reveals that BPharm Student 3 did not participate at all in the second week yet Students 4 and 5 were digitally active in their team on days outside of the required one day a week expectation. Note that the fourth week was the student vacation, so no activity was anticipated but some students were still active online (BPharm Student 5, Figure 9).

The Power Virtual Agents for Teams provided additional data analytics on students’ interactivity with the chatbots, during and after their clinical case and pharmaceutical care planning consultations. Access to, and outputs from, the Power Virtual Agents looked similar to Teams Insights. An array of analytical measures was tracked, including the number of sessions, engagement rates, engagement over time and session outcomes over time. Number of sessions allowed the management team to determine quickly whether each team had completed their chatbot inquiries. The engagement rate relied on participants triggering specific topics within the session. The escalation rate summarized the sessions in which the chatbot referred the students to a human interaction with academic staff, in the role of simulated patient or healthcare professional. This would occur if the student query could not be answered by the chatbot. The analytics allowed tracking of which student questions were asked and where important queries had been missed. Any recognized deficiencies were then fed back to individual students or their teams.

#### 3.2.1. Student Feedback on Hybrid Delivery

In the final debriefing, students reported positively on many aspects of their game experience, stressing their need to be flexible and resilient to meet the challenges of their learning during the pandemic. Anonymous student feedback on the hybrid simulation was obtained from free text comments on course evaluation reports for the semester that delivered PharmG.

Positive exemplar comments include:

*Pharm G is a good activity that helps people work together and understand what it is like in a team. I think that the repetitive MS forms helps massively, giving more structure to how to present during osces [sic] and lets us know what is required during counselling.*

(Student C, Student Evaluation of Course 2021)

*PharmG is the best aspect of this course, and doing it once a week appeals to me better than all at once (in three weeks).*

(Student G, Student Evaluation of Course 2021)

Negative exemplar comments did not often relate to the mode of delivery but included:

*Pharm G is quite like real life however, I think that there was not a lot of consequences individually for failure. I didn’t enjoy having no feedback on the cases.*

(Student S, Student Evaluation of Course 2021)

One negative student comment on the hybrid delivery suggested that some students were not fully using the Teams communication platform that was created for them:

*The barrier in communication between team members in-person and online was hard to overcome, so maybe some communication through Teams can be investigated next year? We wereusing Facebook messenger and messages would be missed and lost in the storm of messages.*

(Student K, Student Evaluation of Course 2021)

#### 3.2.2. Staff Reflection on Hybrid Delivery

The workload required to prepare the gamified simulation in the second year was much reduced but still significant. Staff and students collectively enjoyed the hybrid delivery, which provided more flexibility, variety and opportunities for face-to-face cases. However, managing both in-person and online students simultaneously was somewhat challenging, and there appeared to be more ad hoc reports from teams that some students were contributing less than they should. Connecting with both online and in-person students was enhanced by having at least one staff member operating in the virtual space at all times.

Response rates to student evaluations of the courses that hosted PharmG improved somewhat in 2021 (29.0% and 31.8%) but were below pre-pandemic response rates (61.9% and 69.0% in 2019). The course evaluations also improved over 2020 (4.2 and 4.7 out of 5), indicating that the hybrid delivery was more acceptable to students than the virtual.

## 4. Summary of Technologies Employed

The various approaches and technologies utilized (Figure 1) were strongly influenced by the University’s available resources and subscriptions. Hence, this paper does not intend to compare available resources but reports on the ways in which the Microsoft and Big Interview software and platforms were used, and sometimes repurposed, for the virtualization of gamified simulation.

Table 1 summarizes the technologies employed and some of the benefits and limitations observed by staff and students in this context, at this university, given the institutional licensing agreement. Microsoft Teams and Forms were familiar to most staff and students and were not difficult to manage. In contrast, Microsoft Stream was less familiar to many students and posed some challenges with access and video sharing permissions. Big Interview was new to most staff and students but staff creation of cases in Big Interview was straightforward. Likewise, student access was simple, involving a web link and a unique access code for each case. The more challenging technologies used were those of the Microsoft Power Platform.

## 5. Discussion

The PharmG gamified simulation was effectively virtualized to deliver both fully virtual and hybrid formats of the activity in the first two years of the COVID-19 pandemic. All activities were able to be virtualized in some format so that intended learning outcomes of each activity could be met. While there are growing accounts of the many educational strategies and learning technologies employed in pharmacy education during the COVID-19 pandemic [11,12,27], this paper appears to be the first to describe the virtualization of a complex capstone activity such as the PharmG gamified simulation.

A core learning outcome of the gamified simulation is the development of teamwork and collaboration, which are essential to contemporary pharmacist practice [3]. The virtualized simulation replicated the separate and competitive pharmacy teams of the initial in-person activity, each with their own communication channel. This encouraged and supported student collaboration, with students reporting positively on the development of strong teamwork bonds and collaborations during their respective 2020 and 2021 debriefings. Furthermore, the hybrid delivery in 2021 provided more unique opportunities for in-person collaboration, allowing multiple students from the same team to participate in live, synchronous cases together. At times, they also brought their virtual colleagues into the cases, using mobile devices.

The authenticity of learning activities in pharmacy education supports the essential development of professional values and identity [28]. Previous research into the PharmG simulation that examined participants’ reflective journal entries explored experiential learning and supported the outcome of professional growth [9]. While it is acknowledged that some authenticity of activities and consultations was diluted in the online and hybrid formats, every effort was made to maintain as much authenticity in patient and health professional consultations as could be managed. In fact, the numerous telehealth consultations that student teams experienced mirrored what was occurring in real-world pharmacy practice everywhere throughout the pandemic [29]. Pharmacists in all areas of practice were required to adopt telehealth strategies to replace former in-person practices [30]. This required increased fluency with communication technologies, which enhanced individuals’ digital capability. However, it was evident that the array of software applications and communication tools provided to student teams was not always used as expected, with some students resorting to more familiar social media channels of communication.

The use of Microsoft Teams as the primary platform in both the virtual and hybrid iterations of the PharmG simulation was invaluable. The Teams site allowed for creation of bespoke channels for pharmacy student teams and staff, the Power Apps interface and the Virtual Power Agents chatbots. Staff and students’ familiarity and comfort with Teams increased across the duration of each iteration of the simulation. However, the game platform utilized required students and staff to jump between different Microsoft applications. In contrast, the Microsoft Power Platform has demonstrated capability to deliver the entire game effectively, negating the need for separate applications. The development of a single Power App, which displays game content, records interactions and allows for an AI-assisted chatbot interface (Microsoft QnA Maker) [31] could be sufficient to obviate the need for multiple applications. Information delivered and recorded in the Power App could be securely stored using Microsoft Dataverse [32] and could be displayed for review and marking within password-protected screens in the developed game platform. The benefits of developing and deploying a single Power App application would simplify game management and provide considerable time and cost savings. Deployment of the Power App directly into the Microsoft Team environment has proved to be a simple way to connect communication tools to the game platform.

In addition to the frequent shifts between applications, the ubiquitous use of Forms, for pharmacist or academic marking of verbal counselling and OSCEs, and for student teams completing prescription assessment and dispensing, resulted in large volumes of Excel spreadsheets to be exported from Forms on every day of the simulation. Whilst not difficult, this presented a time burden on the management team during virtualization that did not exist prior to the pandemic. The numerous Excel dispensing templates required additional formatting by the management staff to facilitate external marking of templates and written medicine counselling by colleagues. The advantage of hosting the management team’s headquarters in a unique channel in Teams was that all marking spreadsheets could be stored in a central location and marks entered into the live-updated master spreadsheet, with ongoing edits visible to all of the supervisory team. While virtualized data management posed some minor challenges, the use of Power Apps connected to Dataverse for Teams has the potential to simplify the task.

Prior to the pandemic, verbal medicines counselling was audio-recorded using mobile devices, such as cellular phones. During the virtual and hybrid simulations however, the use of Stream for complete audio and video recording of patient medicines counselling enhanced the authenticity of the activity. In contrast, while the Big Interview platform allowed for asynchronous virtual OSCEs to be undertaken during the pandemic, students observed that the one-sided nature of the consultations lost some of the real-world authenticity of live and synchronous patient interactions. It was recommended that in future learning and teaching, Big Interview might be better suited to junior pharmacy students, providing them an avenue to self-reflect on and self-correct their own patient communication skills safely [20]. This has since been adopted, and Big Interview now serves as the key platform for both formative and summative asynchronous OSCEs for first and second year pharmacy students enrolled in the Griffith University Bachelor of Pharmacy degree program.

### Strength and Limitations

The Griffith University PharmG team was able to virtualize the simulation using a multitude of Office 365 applications available in the institutional license. However, institutions that do not have access to Office 365, or that use different technological applications, may be unable to replicate this approach. The team was strengthened by members’ interest in, and fluency with, digital learning technologies. This was further supported by people’s willingness to learn new approaches and enhance their digital capability under the time pressure brought on by the pandemic.

In addition to limitations discussed above, the Teams analytics currently have limitations in that data are only readily accessible in standard settings for 90 days. While this allows for timely monitoring of student participation and engagement during the activity at hand, opportunities for later data analysis or research are currently restricted.

## 6. Conclusions

The successful virtualization of the Australian gamified simulation PharmG in the first two years of the COVID-19 pandemic utilized a range of digital technologies to support and deliver the important learning outcomes of the activity. Student participants were required to be flexible, responsive and resilient, which prepared them well for real-world pharmacist practice during a global pandemic.

## Figures and Tables

**Table 1:** Summary of Technologies Employed.

Software Platform	Benefits	Limitations
Microsoft Teams	Main platform for pharmacy team channels, communication and file management; private channels available; accessibility friendly, e.g., transcripts available	Subscription required as part of Microsoft Office 365
Microsoft Stream	Asynchronous video recording for verbal counselling	Video sharing permission issues complicated marker access
Microsoft Forms	Replicable templates created easily for student use, e.g., dispensing counselling, and staff use for marking	Outputs involve many Excel files to manage; manual mark calculation required due to mark allocation and calculation limitations in Forms
Microsoft Power Apps	Game-like interface to virtual pharmacies in Microsoft Teams	Required technologically adept staff to develop, share, deploy and maintain
Microsoft Power Virtual Agent	Artificial intelligence chatbots allowed students to ask natural language questions of virtual patients or prescribers	Required technologically adept staff to develop and insights into sharing and deployment
Big Interview	Asynchronous video recording for OSCEs or counselling, in response to patient scenarios	Lack of patient response; loss of authenticity; some student difficulties in getting videos to upload or save; slow video loading delayed marker access
